# Mei-P26 bidirectionally modulates feeding behavior, locomotion and glucose levels via insulin-neurons in *Drosophila*

**DOI:** 10.17912/micropub.biology.002100

**Published:** 2026-06-03

**Authors:** Tolulope Olaolorun, Elena Pak, Alexander Murashov

**Affiliations:** 1 Comparative Biomedical Sciences, Louisiana State University, Baton Rouge, LA, US

## Abstract

*Mei-P26* is associated with cell-fate regulation in *Drosophila melanogaster* . In prior work, offspring of Western diet-fed fathers exhibited hyperphagia accompanied by increased brain *Mei-P26.* Because feeding is regulated by insulin/ILP signaling, we tested whether altering *mei-p26* expression in insulin-producing cells affects behavioral and metabolic outcomes. Here we show that overexpression of *mei-p26* in ILP2 neurons reduced feeding (FLIC), consumption (ConEx), locomotor activity, and glucose levels by 30-50%, whereas *mei-p26* knockdown produced opposite effects. These effects were consistent across sexes, identifying *Mei-P26* as a potential regulator within insulin-producing cells associated with bidirectional changes in adult behavioral and metabolic readouts.

**Figure 1. ILP2-specific manipulation of Meip26 alters glucose homeostasis, locomotor activity, and feeding behavior in adult flies:** Whole-fly glucose levels, locomotor activity, feeding events, and total food consumption were assessed in adult male and female flies following ILP2-specific overexpression (OE) or knockdown (KD) of Mei-p26.(A-B) Locomotor activity counts in females (A) and males (B) expressing ILP2-driven Mei-p26 overexpression show reduced activity relative to controls. “Counts” represent infrared beam breaks recorded by the *Drosophila* Activity Monitoring (DAM) system and are expressed as counts per 30-min interval, serving as a quantitative measure of fly movement(C-D) Locomotor activity is increased in females (C) and males (D) expressing ILP2-driven Mei-p26 RNAi.(E-F) Feeding events measured by the Fly Liquid-Food Interaction Counter (FLIC) are increased in females (E) and males (F) upon Mei-p26 knockdown in ILP2 neurons. An “event” is defined as a discrete physical interaction between the fly proboscis and the liquid food source that closes an electrical circuit, serving as a quantitative proxy for feeding behavior.(G-H) Feeding events following ILP2-specific Mei-p26 overexpression are significantly reduced in females (G) and males (H) relative to controls.(I-J) Total food consumption assessed by the Consumption and Excretion (ConEx) assay is increased in males (I) and females (J) expressing Mei-p26 RNAi under control-diet conditions.(K-L) Total food consumption is reduced in females (K) and males (L) expressing Mei-p26 overexpression.(M-N) Whole-fly glucose levels are increased following ILP2-driven Mei-p26 knockdown when normalized to total protein content in females (M), and males (N).(O-P) Whole-fly glucose levels are reduced in females and males expressing ILP2-driven Mei-p26 overexpression when normalized to total protein content For locomotor assays, *n* = 20 flies per group. For FLIC assays, *n* = 12 flies per group. For ConEx assays, *n* = 8 biological replicates per group, with 15 flies per replicate. For glucose measurements, each biological replicate consisted of 5 flies, with 10 biological replicates per group. Flies were homogenized and glucose levels were quantified using a colorimetric glucose assay (Horiba Glucose Assay Kit, POINTE Scientific). Glucose values were normalized to mean fly weight and/or total protein content as indicated. Bars represent mean values with individual data points shown; error bars indicate SEM. Statistical significance was assessed using unpaired two-tailed Student’s or Welch’s *t* -tests where appropriate. *p* < 0.05, p < 0.01, *p* < 0.001, p < 0.0001; n.s., not significant.

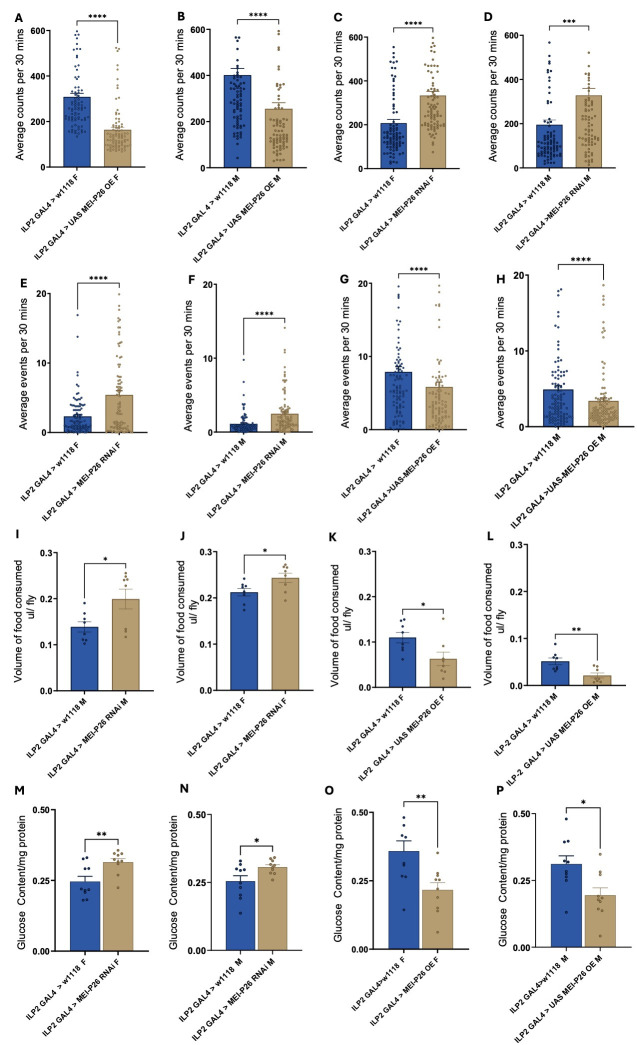



## Description

Drosophila insulin-like peptides (DILPs) are evolutionarily conserved regulators of growth, metabolism, and nutrient homeostasis. In adult flies, the major circulating DILPs (DILP2, DILP3, and DILP5) are produced and secreted by a small cluster of neuroendocrine insulin-producing cells (IPCs) located in the pars intercerebralis of the brain, functionally analogous to mammalian pancreatic β-cells (Brogiolo et al., 2001; Rulifson et al., 2002; Nässel & Vanden Broeck, 2016). These peptides signal through the *Drosophila* insulin receptor to regulate systemic carbohydrate metabolism, energy storage, growth, lifespan, and feeding behavior (Broughton et al., 2005; Grönke et al., 2010). IPC activity is dynamically modulated by nutritional state and circulating metabolites, allowing rapid adjustment of peptide synthesis and secretion in response to dietary inputs (Manière et al., 2016). Among the DILPs, DILP2 is one of the predominant insulin-like peptides in adult IPCs and plays a central role in coordinating feeding and metabolic homeostasis.

Genetic or physiological perturbations of IPC function alter food intake and metabolic balance, underscoring the sensitivity of this neuroendocrine axis to regulatory inputs (Broughton et al., 2005; Grönke et al., 2010). While upstream signaling pathways governing IPC development and ILP secretion have been extensively characterized, comparatively less is known about how intracellular post-transcriptional regulators shape IPC-mediated physiological outputs in adult animals.

In previous work, we reported that offspring derived from fathers exposed to a Western diet for five days displayed a hyperphagic phenotype accompanied by elevated levels of the protein Mei-p26 in the brain (Murashov et al., 2023). Mei-p26 is a conserved TRIM-NHL family RNA-binding protein with established roles in translational regulation and cell-state control in the *Drosophila* germline (Insco et al., 2009; Insco et al., 2012; Neumüller et al., 2008). Despite this extensive characterization in developmental contexts, whether Mei-p26 contributes to physiological regulation in adult neuroendocrine neurons has not been examined.

Given the established role of IPCs and ILP2-expressing neurons in feeding regulation and systemic metabolic control (Broughton et al., 2005; Grönke et al., 2010), we investigated whether altering Mei-p26 levels specifically in ILP2 neurons affects behavioral and metabolic outputs in adult flies. To this end, we performed ILP2-specific overexpression (OE) and RNAi-mediated knockdown (KD) of Mei-p26 and quantified locomotor activity, feeding behavior, total food consumption, and whole-fly glucose levels in adult male and female flies.

Locomotor activity was measured using the *Drosophila* Activity Monitoring (DAM) system. ILP2-specific overexpression of Mei-p26 significantly reduced activity counts per 30-minute interval in females ( Figure 1A ) and males ( Figure 1B ) relative to matched controls. Conversely, ILP2-specific knockdown increased locomotor activity in females ( Figure 1C ) and males ( Figure 1D ), demonstrating bidirectional modulation of activity by Mei-p26 in ILP2 neurons. Feeding behavior was assessed using the Fly Liquid-Food Interaction Counter (FLIC), which quantifies discrete proboscis-food contacts as feeding events (Ro et al., 2014). ILP2-specific knockdown of Mei-p26 significantly increased feeding events in females ( Figure 1E ) and males ( Figure 1F ). In contrast, ILP2-driven overexpression reduced feeding events in females ( Figure 1G ) and males ( Figure 1H ).

Total food intake was further quantified using the Consumption-Excretion (ConEx) assay (Shell et al., 2018). Mei-p26 knockdown increased total food consumption in males ( Figure 1I ) and females ( Figure 1K ), whereas overexpression reduced consumption in females ( Figure 1J ) and males ( Figure 1L ). These findings are consistent with the FLIC-derived feeding event measurements. Whole-fly glucose levels were measured and normalized to total protein content. Mei-p26 knockdown increased glucose levels in males ( Figure 1N ), and females ( Figure 1M ). Conversely, ILP2-specific overexpression reduced glucose levels in females ( Figure 1O ) and males ( Figure 1P ).

Across independent behavioral and metabolic assays, ILP2-specific manipulation of Mei-p26 produced reproducible effects: overexpression was associated with reduced locomotor activity, reduced feeding, and lower glucose levels, whereas knockdown resulted in increased activity and feeding, with corresponding elevations in glucose levels in males. Together, these data demonstrate that altering Mei-p26 levels within ILP2-expressing neurons is sufficient to modulate IPC-associated behavioral and metabolic outputs in adult flies.

## Methods

Fly Husbandry

All flies were maintained on standard cornmeal-yeast-agar medium under a 12:12 h light-dark cycle at 25 °C. Crosses were established using ILP2-GAL4 driver lines and UAS- *mei-* P26 overexpression or UAS- *mei* - *P26* RNAi lines. Parental crosses were maintained at 25 °C, and adult progeny were collected within 24 h of eclosion and aged to 5 days prior to behavioral and metabolic assays. Males and females were collected and analyzed separately. All assays were performed using age-matched flies. Stocks obtained from the Bloomington Drosophila Stock Center (NIH P40OD018537) and the Vienna Drosophila Resource Center (VDRC) were used in this study. The following fly lines were used: *w* ^1118^ (BDSC_3605), ILP2-GAL4 (BDSC_37516), *UAS-mei-P26* (BDSC_25771), mei-P26 RNAi (VDRC_101060).

Genetic Crosses

To manipulate Mei-p26 levels in insulin-producing cells, ILP2-GAL4 was used to drive either overexpression (OE) or RNAi-mediated knockdown (KD) of Mei-p26 in ILP2-expressing neurons. OE and KD experiments were conducted as independent experimental series, and each genotype was compared to its genetic control (ILP2-GAL4 × w1118) generated and assayed in parallel.

Feeding Behavior: Fly Liquid-Food Interaction Counter (FLIC)

Feeding behavior was quantified using the Fly Liquid-Food Interaction Counter (FLIC). Individual age-matched 4-5-day-old flies were loaded into FLIC chambers containing liquid food, and feeding events were recorded continuously for 36-48 h. An event was defined as a discrete proboscis-food contact sufficient to close the electrical circuit, serving as a quantitative proxy for feeding behavior. Data was extracted as total feeding events per fly and normalized per 30-minute intervals. Flies were maintained under controlled environmental conditions during recording.

Food Consumption: Consumption-Excretion (ConEx) Assay

Total food intake was measured using the Consumption-Excretion (ConEx) assay. Groups of age-matched 4-5-day-old flies were transferred to blue dye-labeled (control) or red dye-labeled western diet for 18 h under standard conditions. Following exposure, excreted dye was quantified using UV-Vis spectrophotometry to estimate total consumption. Intake values were recorded and each biological replicate consisted of a defined group of flies assayed in parallel under identical conditions.

Locomotor Activity Monitoring (DAM System)

Locomotor activity was measured using the Drosophila Activity Monitoring (DAM) system (Trikinetics). Individual age-matched 4-5-day-old flies were placed in glass monitoring tubes containing standard food, and infrared beam breaks were recorded as activity counts. Activity was analyzed as average counts per 30-minute interval over the recording period. All recordings were performed under controlled light-dark conditions to minimize circadian variability.

Whole-Fly Glucose Measurement

Whole-fly glucose assays were carried out as described previously (Tennessen et al., 2014). Briefly, groups of five age-matched 4-5-day-old flies were rapidly homogenized in 200 µL of assay buffer using 0.5-1 mm zirconium beads in a Bullet Blender homogenizer (Next Advance, Inc., Troy, NY). The samples were centrifuged for 10 min at 10,000 × g at 4 °C to pellet cellular debris. Total protein levels were measured using the Pierce Rapid Gold BCA Protein Assay Kit (A55860, Thermo Fisher Scientific). Glucose measurements were performed using the Horiba Glucose Assay Kit (G7521-500, POINTE Scientific) and quantified on a SpectraMax plate reader. Glucose levels were normalized to total protein content.

Statistical Analysis

All statistical analyses were performed using GraphPad Prism version 10.0. For each experimental series (OE and KD). Data are presented as mean ± SEM with individual data points shown. Statistical significance between two groups was assessed using unpaired two-tailed Student’s t-tests or Welch’s t-tests where variance differed between groups. A significance threshold of p < 0.05 was applied. 
